# Notes and News

**Published:** 1891-08-01

**Authors:** 


### Notes and News.

Collected and Communicated.

The new Epidemic Hospital for Nottingham, at Bagthorpe, is now completed, and has been formally opened. The cost of the building, exclusive of site, was £25,000. At present accommodation for eighty-two patients has been provided. The building is after the designs of the borough engineer, Mr. Arthur Brown, and all the most modern appliances and fittings have been adopted.

The successor to Dr. Koch at the Institute of Hygiene in Berlin has been found in the person of Professor Rubner, of Maiberg. Strange to say, the latter is not Professor Koch's candidate, but a pupil of Professor Pettenkofer's, and the nominee of Professor Virchow. The appointment is a distinct triumph for Virchow's party. The unsuccessful candidate was Professor Flügge, one of Koch's earliest pupils.

The machinery of the law is proverbially unwieldy, and not infrequently defeats its own ends, and citizens adhering too strictly to the letter occasionally come in for a reprimand by ignoring the spirit. Such was recently the case at Cork. A case of fever occurred in a private dwelling, and everyone was so afraid of usurping someone else's prerogative that no order for the disinfection of the premises was given until fifteen days after the patient's removal to the hospital, and subsequent death. The Dispensary Committee had a meeting, and decided that some one was to blame, but owing to technical obstruction, the culprit could not be fixed upon. It was decided to lay down more definite rules for the future.

The Charitable Institutions of Stockton give us examples of what may be done by the working classes to assist themselves when under temporarily adverse circumstances. Greater interest will be felt in the institutions, when the working classes are afforded an opportunity of paying according to their means for the relief they receive at the hospitals. Sir Horace Davey, who addressed the meeting of the Stockton Friendly Societies on Hospital Sunday, referred to the unique character of the Stockton Hospital. He said it was a "hospital of the people, supported by the people, and used by the people." He stated that two-thirds of the income of the hospital was subscribed by the working classes. There is a significant rule in the bye-laws of the nursing association of the Stockton district by which any body of working men subscribing £10 and upwards per annum is entitled to appoint a representative on the Council.

Mr. Waddilove, Chef of University College, Oxford, has presented the Radcliffe Infirmary with a gift of £1,000, to be devoted to the erection of a children's ward in memory of his daughter, who had intended to devote herself to the nursing career. The new ward is to be called the Lizzie Waddilove Ward.

The Secretary of the Richmond Hospital calls attention to a collecting plate, on which is inscribed the name of the institution, which is used by that hospital for the purpose of collecting at places of entertainment. The advantages of a plate over a box as a collecting medium under some circumstances is obvious, and the guarantee which an officially stamped plate affords the more cautious of the public warrants the adoption of such a method.

The fourth annual report of the London Skin Hospital shows that during the past year there were admitted to the institution 1,189 new out-patients, and the attendances numbered 7,601, making a total of 4,286 patients and 24,397 attendances since the establishment of the hospital. There has been a marked increase in the amount of donations and subscriptions, but liabilities still exist to the amount of £465. This deficiency is mainly due to the continued increase of patients and to the removal of the hospital, on June 24th last, to larger premises at 40, Fitzroy Square, W.

It is already a matter of comment and surprise that at the Congress of Hygiene to be held in August, Miss Nightingale should be the only representative of her sex on the published list of Patrons and Council. The matter to be discussed before the Congress embraces subjects to which such ladies as Miss Twining, Miss Octavia Hill, Miss Toynbee, and Mrs. Davenport Hill have devoted a life's work and interest, and upon which they pre-eminently have the right to speak with authority. Does the Congress not profess to be of a representative character, or have these ladies declined to give the meetings the benefit of their experience? In either case both the public and science must suffer.

The report of the Cork Hospital for Women and Children for 1890 is satisfactory. There is an improvement in the financial affairs of the hospital, and the nursing under Miss Baxter, the able Matron, has been admirably conducted. The low cost per bed at this institution should be a subject for reflection to the management of many hospitals. Few can show so low an expenditure as £27 11s. per head. The great want in the hospital seems to be an isolation ward, as an outbreak of scarlatina made it necessary to close the hospital for a time. This would have been unnecessary had the institution possessed an isolation ward distinct from the main building, as all children's hospitals should do.

The new Lunacy Acts have now had a fair trial, and they stand almost universally condemned, except, perhaps, by the proprietors of private asylums. Already a new Bill to amend these Acts has appeared, and on looking over this new creation we are inclined to think that some improvements would be advisable before it becomes an Act. It leaves so much untouched that few would be the worse were it thrown out altogether. Indeed, anything in the shape of amendment to the Acts would seem a hopeless task. An entirely new Bill is required, and we earnestly hope this will soon see the light. In the meantime it will affect the situation very little whether this innocent be slaughtered or tenderly nursed to manhood.

